# Supplementary material for: SMARTer single cell total RNA sequencing
Source: Nucleic Acids Res. 2019 Jun 19;47(16):e93. doi: 10.1093/nar/gkz535 (PMC6895261; doi:10.1093/nar/gkz535)
Supplement: gkz535_Supplemental_Files [file gkz535_supplemental_files.zip › Supplemental1_Figures.pdf]

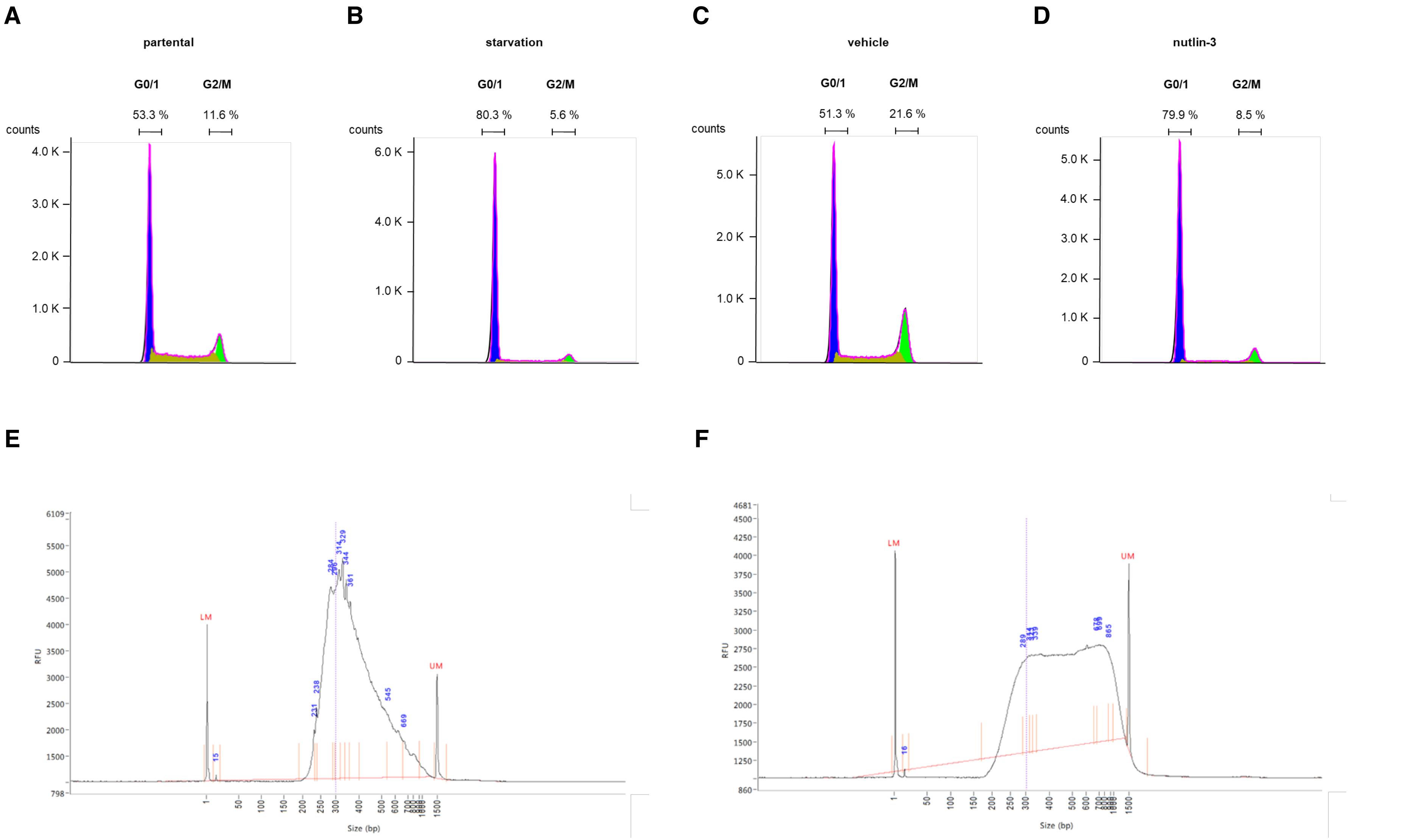

Supplementary Figure 1. Cell cycle profiles for NGP cells maintained in RPMI-1640 medium supplemented with 10 % serum (A) or 0 % serum (B) for 24 hours. Cell cycle profiles for NGP cells maintained in 0 % serum for 24 hours and treated with vehicle (C) or nutlin-3 (D) for 24 hours. Fragment analyzer profiles of a NGP total RNA library (E) and polyA[+] RNA library (F).

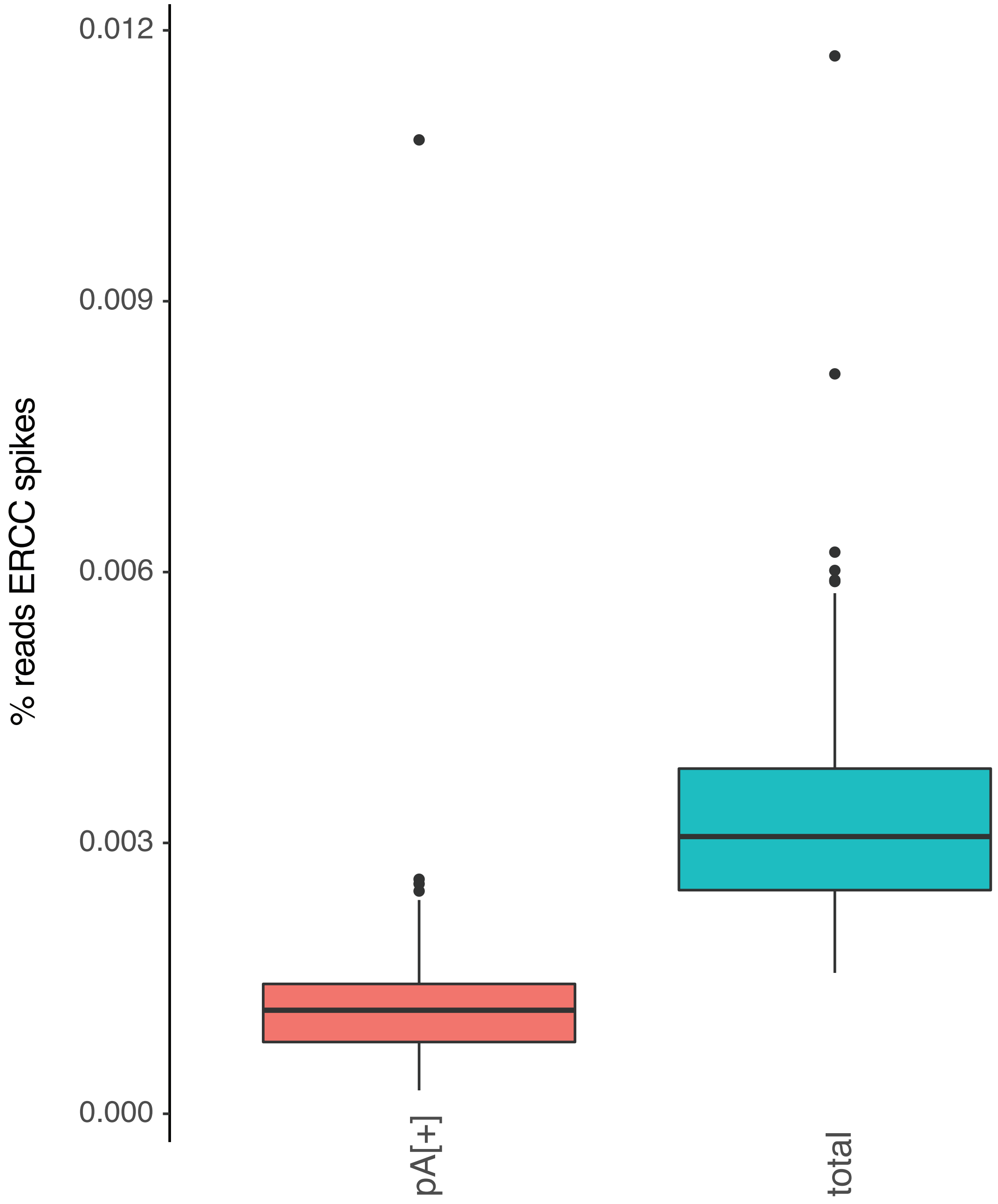

Supplementary Figure 2. The percentage of reads mapped on ERCC spikes is higher in the total RNA libraries compared to polyA[+] RNA libraries.

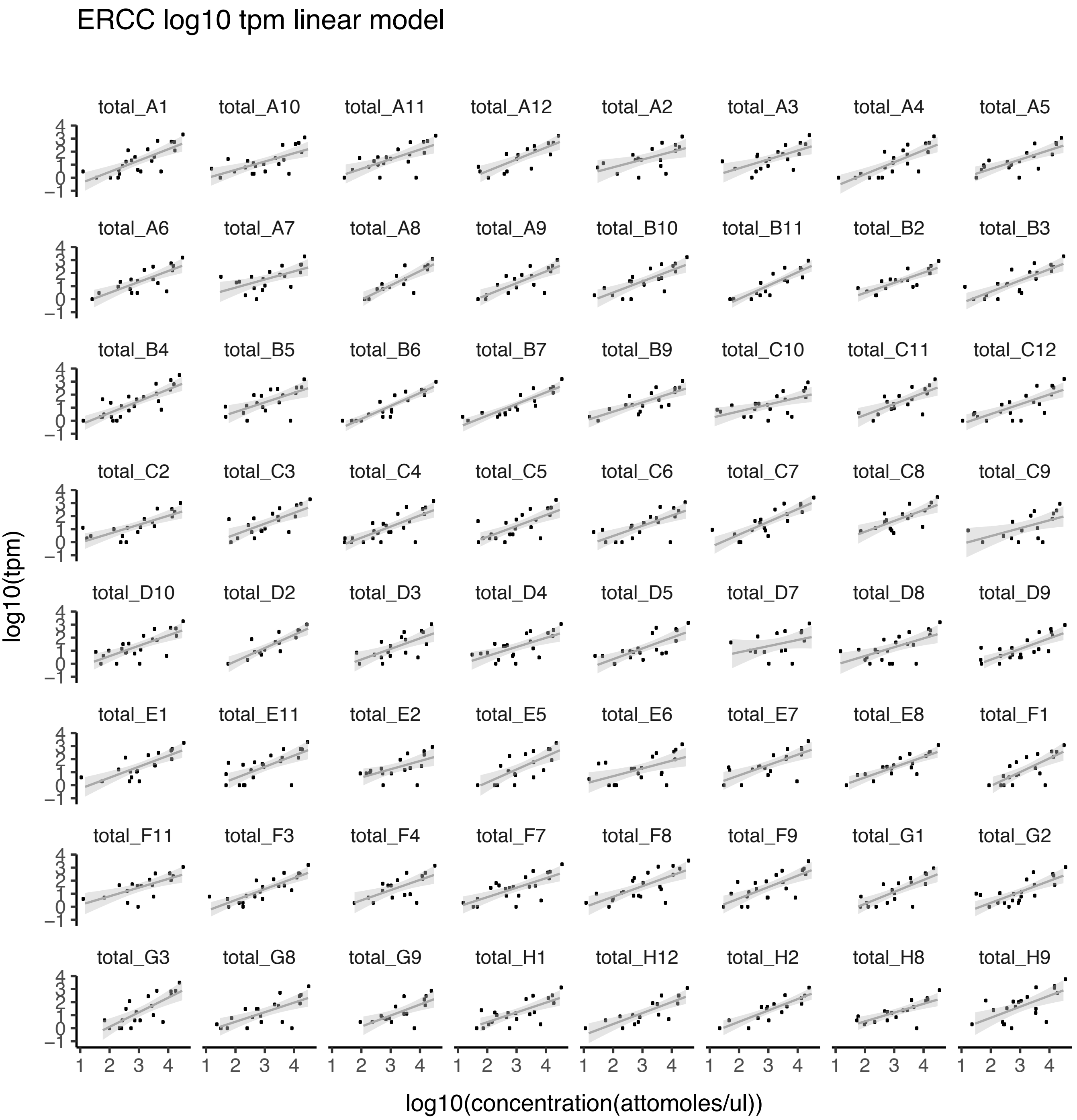

Supplementary Figure 3. Linear modeling of ERCC spike abundance demonstrates quantitative performance.

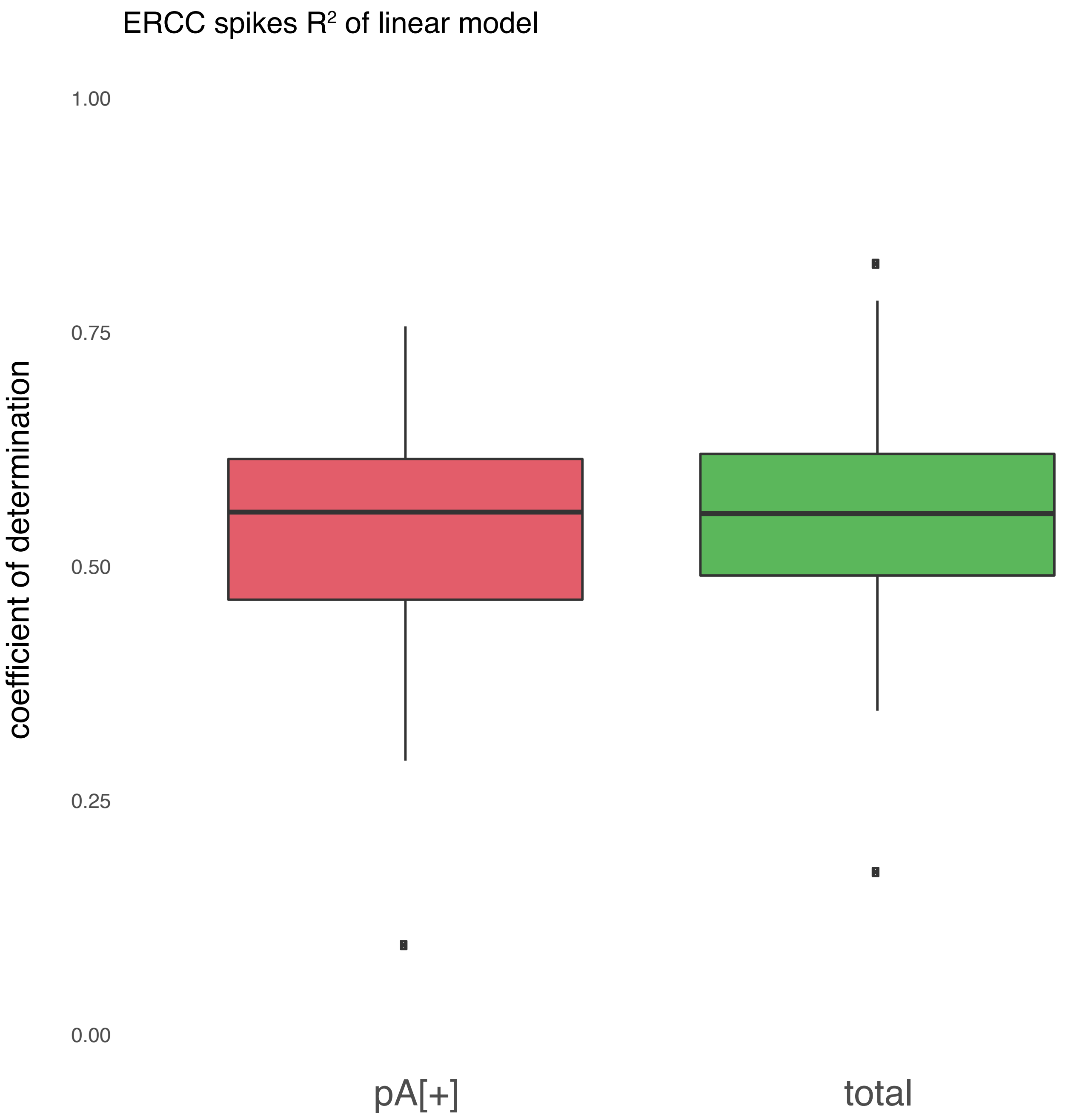

Supplementary Figure 4. The coefficients of determination obtained through linear regression of ERCC spikes are equal for polyA[+] and total RNA libraries.

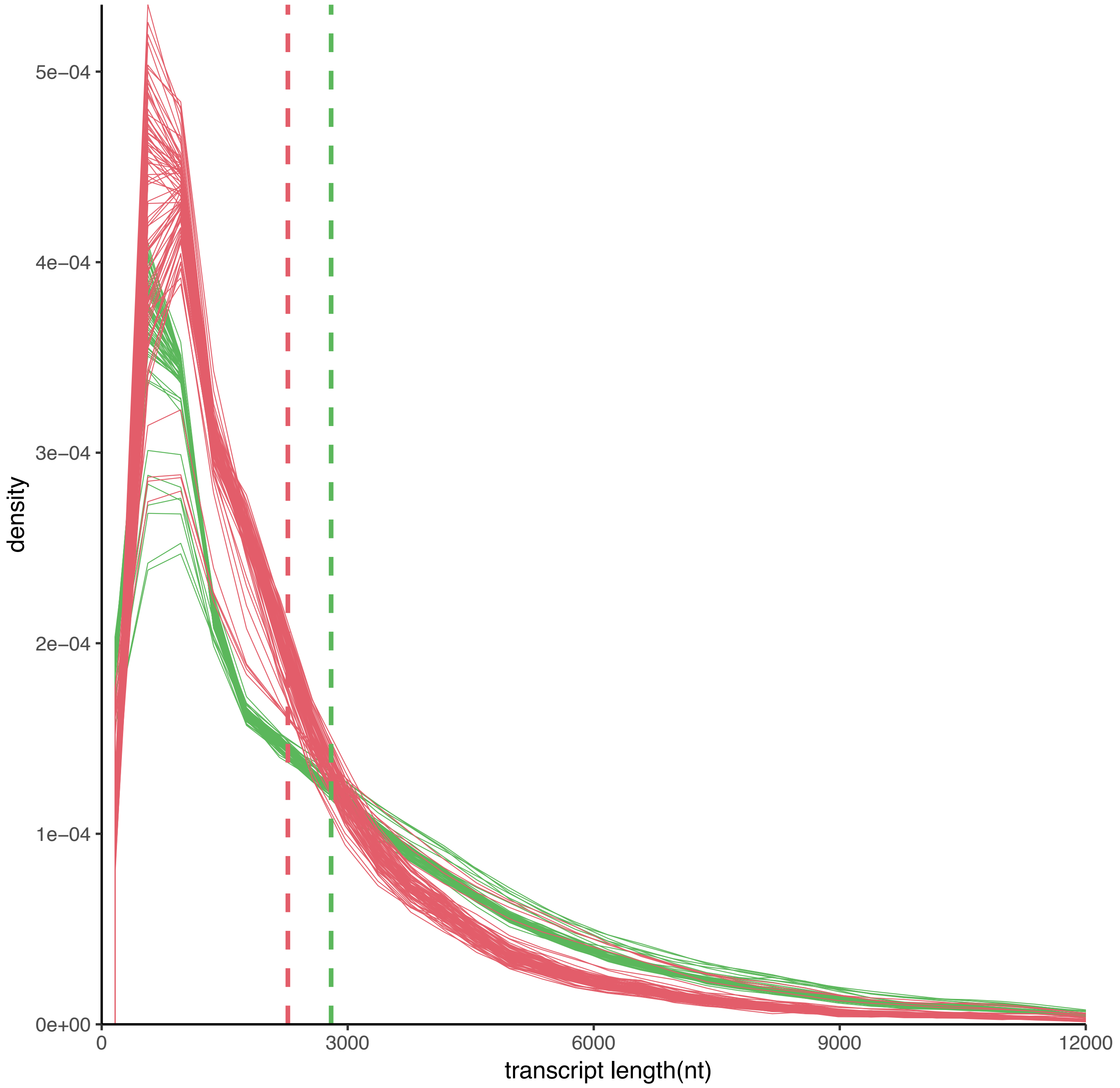

Supplementary Figure 5. The length distributions differ between polyA[+] RNA transcripts (red) and total RNA transcripts (green) per cell. PolyA[+] RNA libraries typically result in the detection of shorter transcripts.

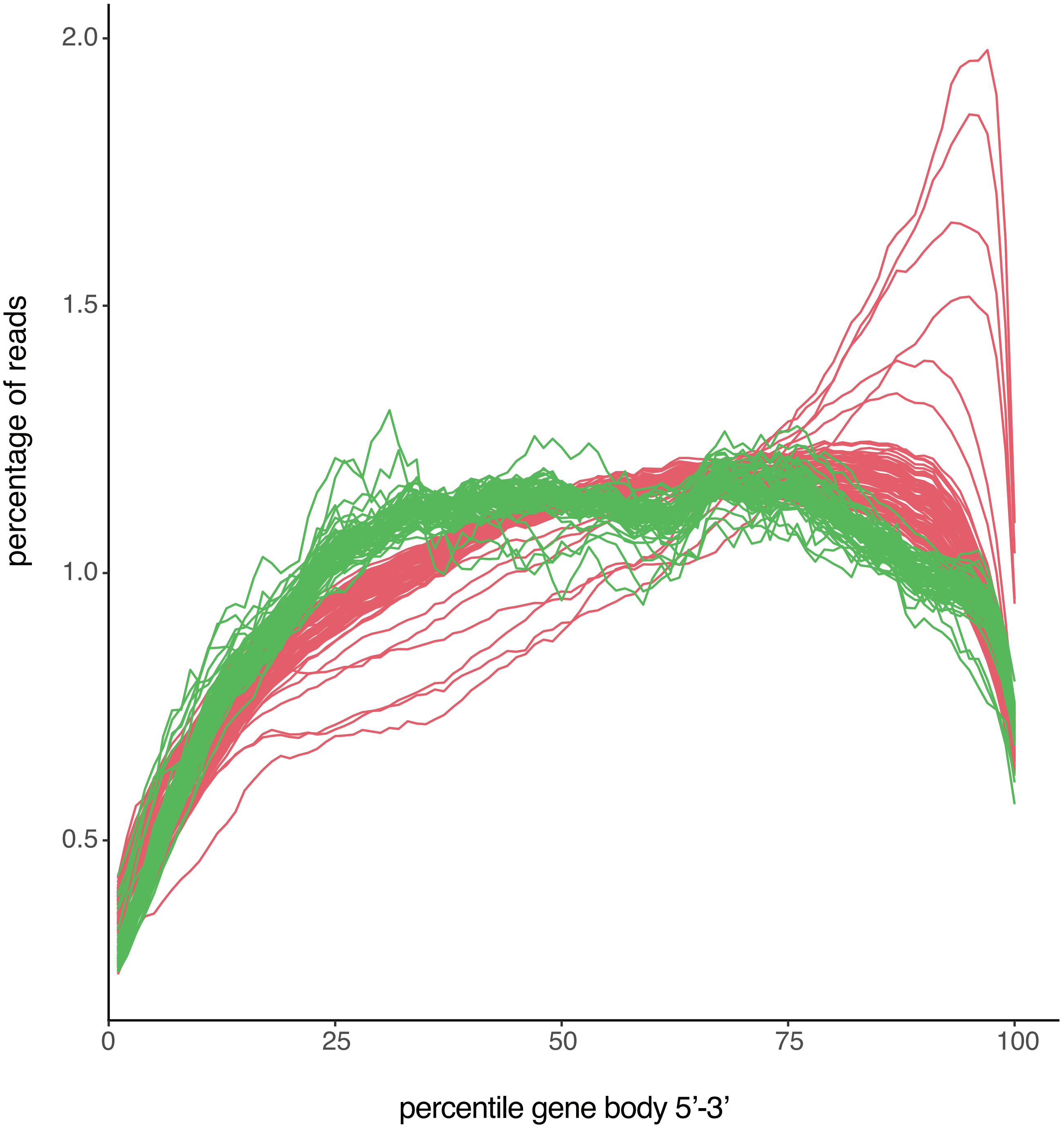

Supplementary Figure 6. The gene body coverage differs slightly between polyA[+] RNA libraries (red) and total RNA libraries (green). Equal gene body coverage would result in 1 % read fraction along the entire gene body. Both library types show some bias towards the 3' and 5' end.

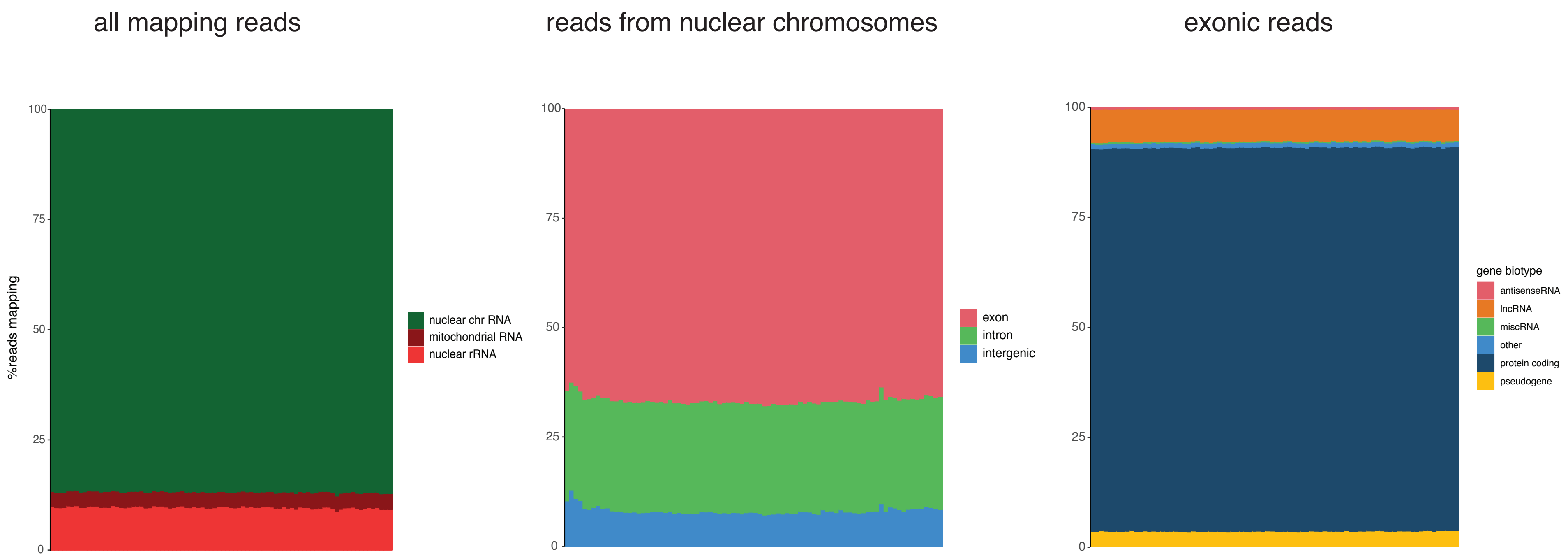

Supplementary Figure 7. The QC results for RAMDA-seq libraries show higher rRNA percentages. A) Percentage of reads derived from nuclear RNA, mitochondrial RNA and ribosomal RNA per cell quantified with STAR. B) Percentage of reads originating from nuclear chromosomes derived from exonic, intronic and intergenic regions per cell quantified with STAR. C) Percentage of exonic reads attributed to the different biotypes per cell quantified with Kallisto.

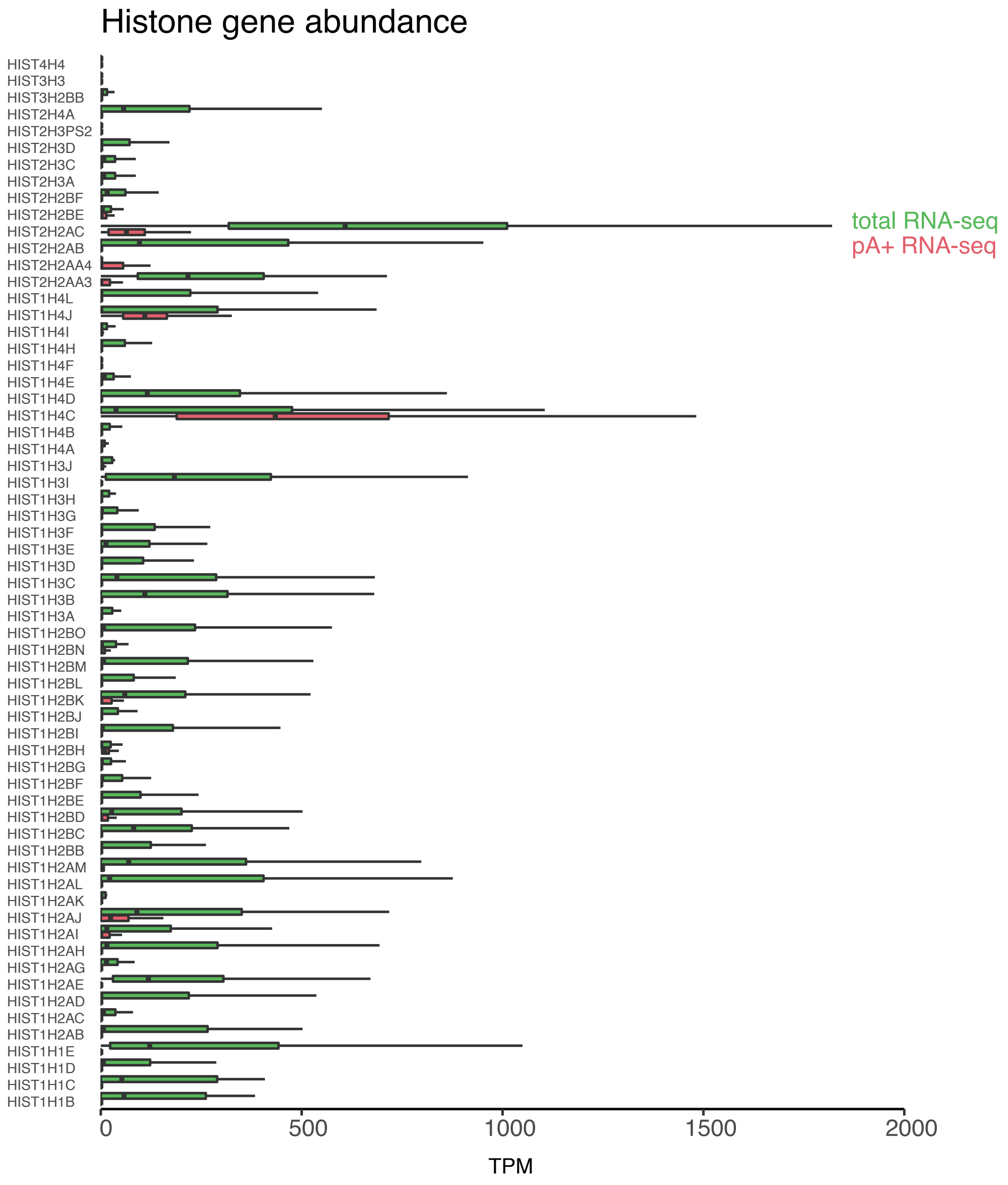

Supplementary Figure 8. TPM expression of histone genes, typically non-polyadenylated, shows that total RNA libraries (green) efficiently capture non-polyadenylated transcripts.

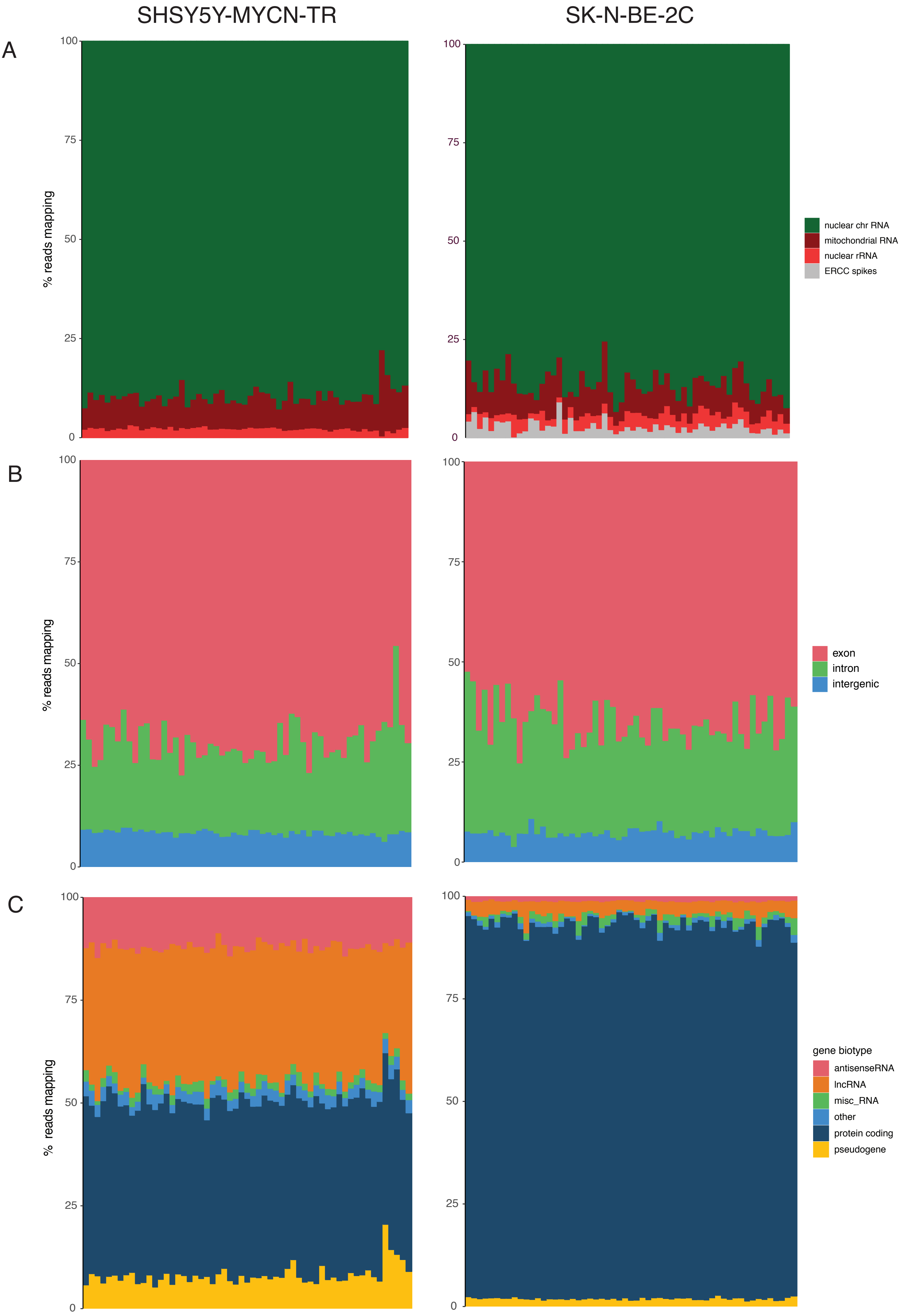
SHSY5Y-MYCN-TR
SK-N-BE-2C
A
B
C
% reads mapping
100
75
50
25
0
nuclear chr RNA
mitochondrial RNA
nuclear rRNA
ERCC spikes
exon
intron
intergenic
gene biotype
antisenseRNA
lncRNA
misc_RNA
other
protein coding
pseudogene

Supplementary Figure 9. The QC results for total RNA libraries of SHSY5Y-MYCN-TR and SK-N-BE-2C cells are similar to NGP cells. A) Percentage of reads derived from nuclear RNA, mitochondrial RNA and ribosomal RNA per cell quantified with STAR. B) Percentage of reads originating from nuclear chromosomes derived from exonic, intronic and intergenic regions per cell quantified with STAR. C) Percentage of exonic reads attributed to the different biotypes per cell quantified with Kallisto.

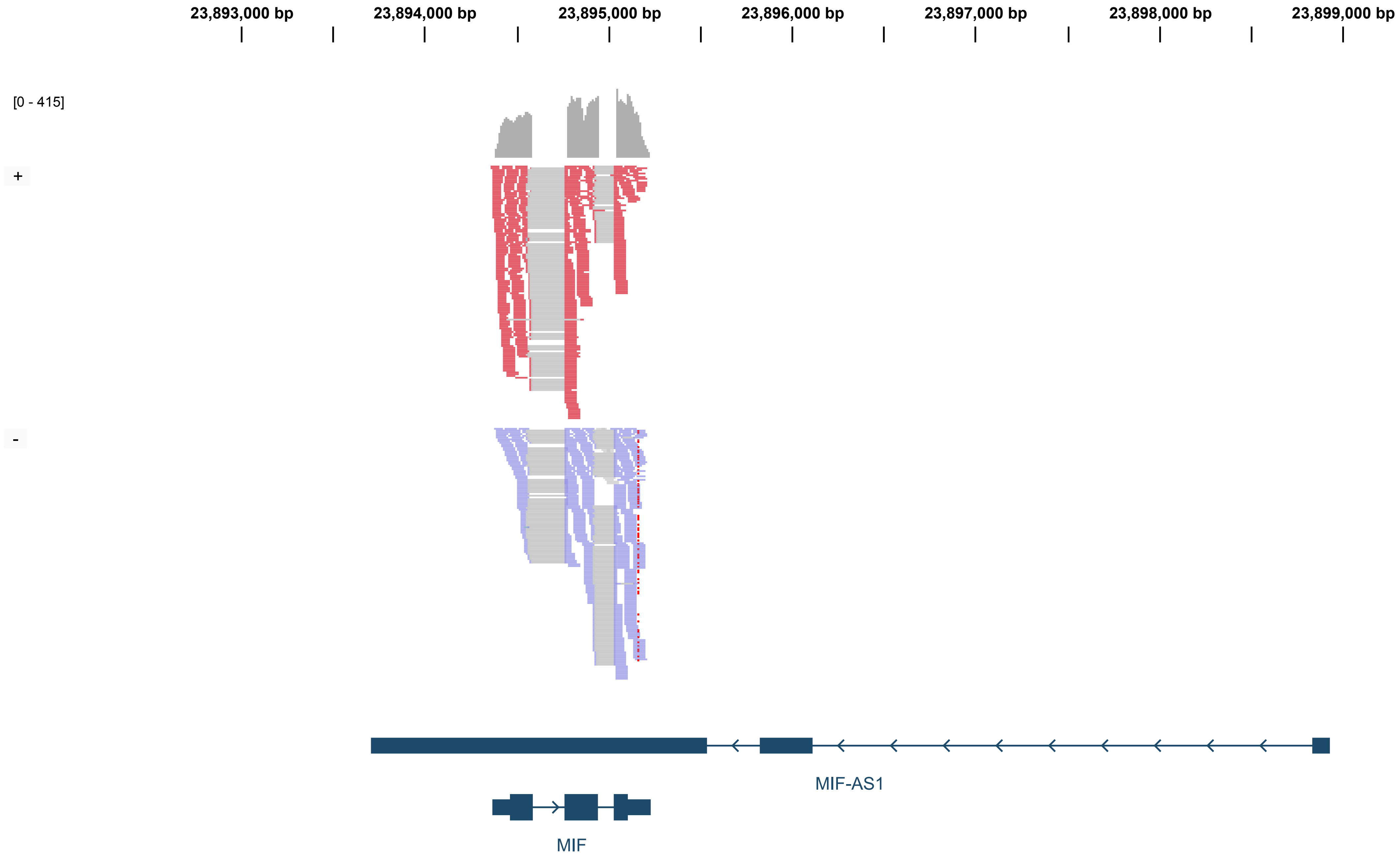

Supplementary Figure 10. IGV visualisation of sense-antisense gene pairs for polyA[+] RNA. The reads mapping on the sense (red) and antisense (blue) strand can not be unambigously assigned to the MIF gene as the data is unstranded. While the counts will be partially mis-assigned to the MIF-AS1 gene, the reads clearly have the splice pattern of only the MIF gene.

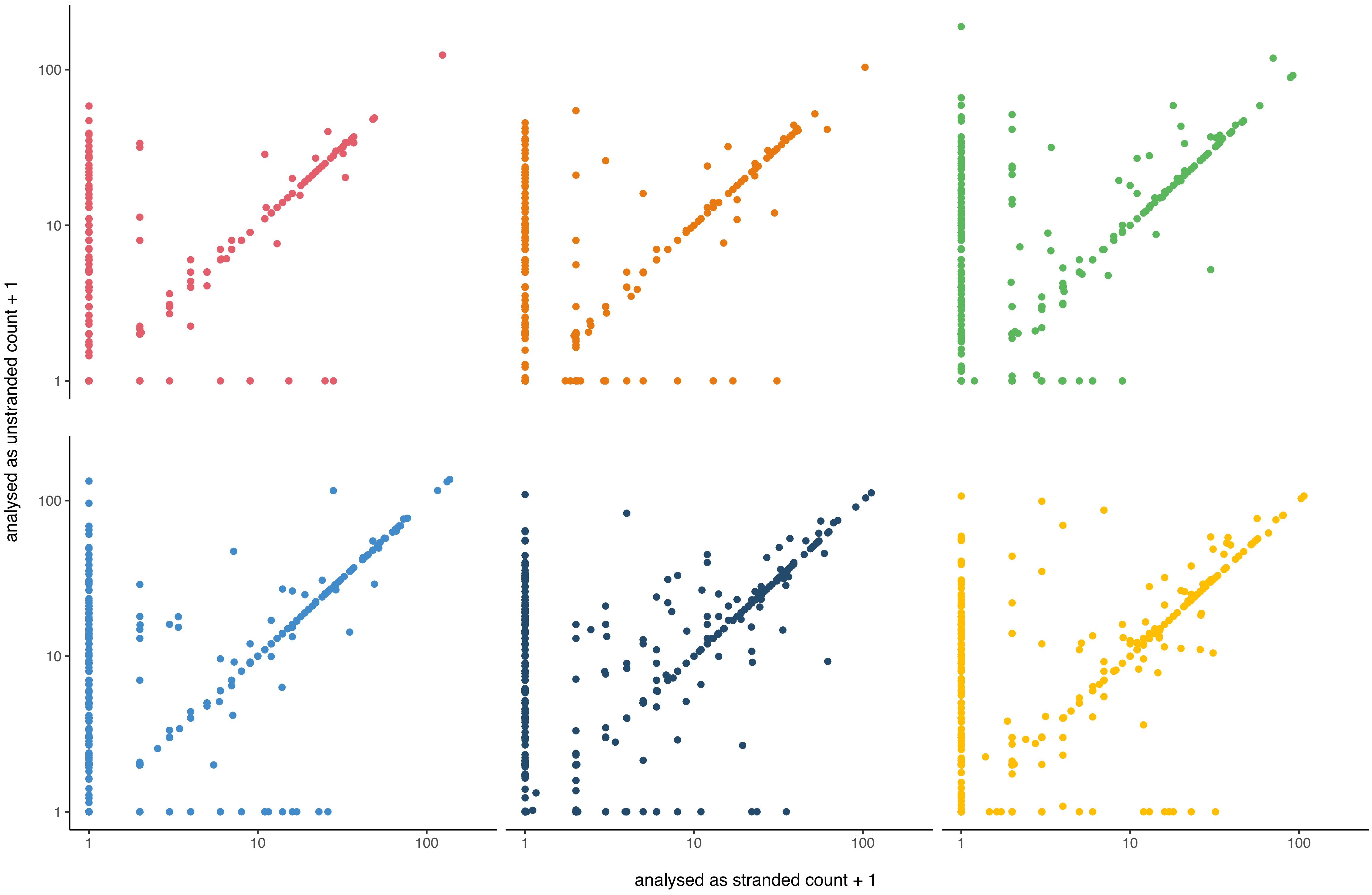

Supplementary Figure 11. Quantification in stranded and unstranded analysis mode of SMARTer total RNA seq data on antisense genes in six randomly selected cells demonstrates substantial misquantification of antisense genes in unstranded mode.

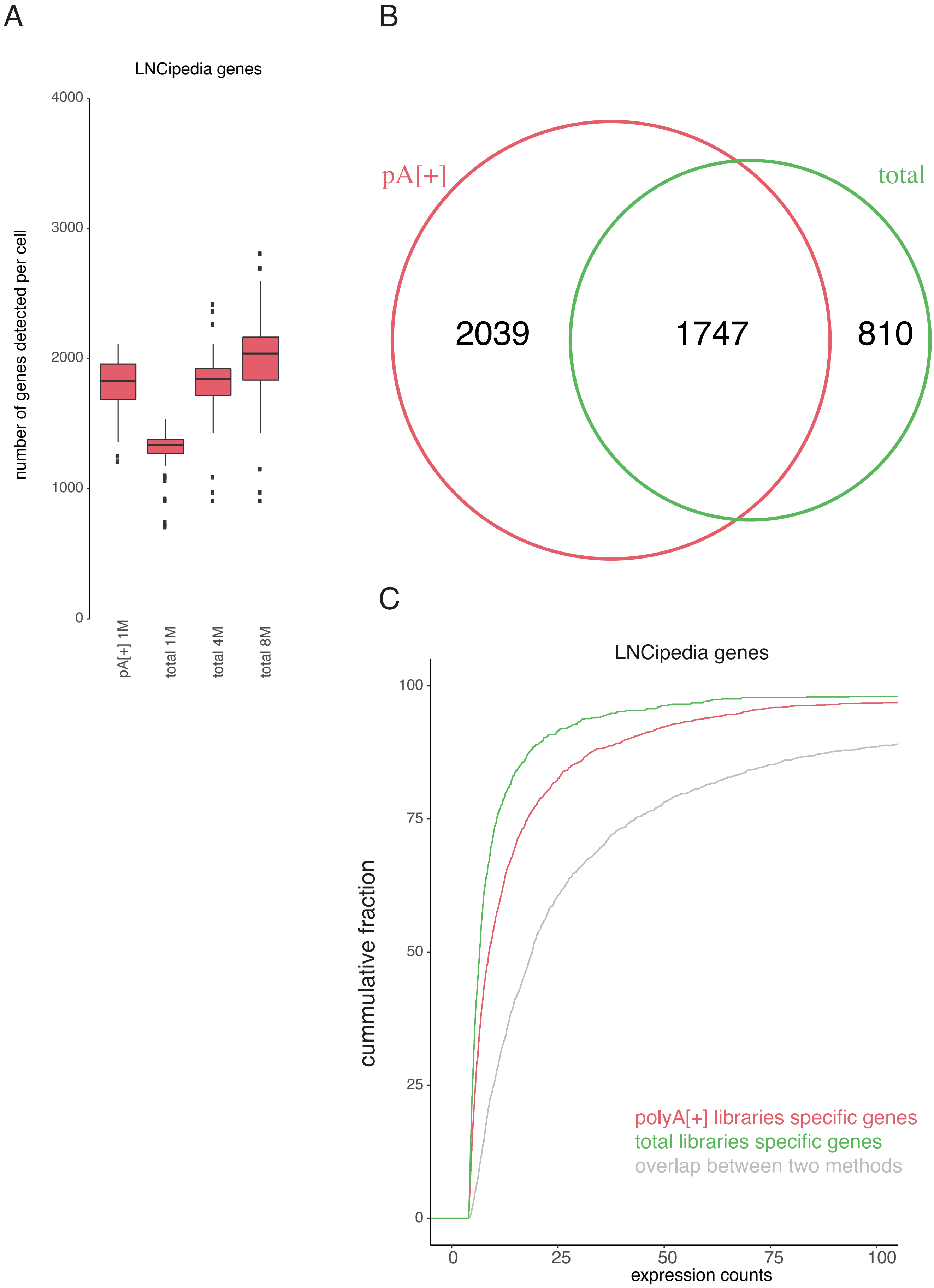

Supplementary Figure 12. By using the LNCipedia transcriptome for quantification, a higher number of lncRNAs was discovered. (A) Number of LNCipedia genes detected in subsampled data (1, 4 and 8 million reads per cell). The proportions are equal compared to Ensembl lncRNAs overlap. (B) Overlap between lncRNAs detected in polyA[+] and total RNA libraries. (C) Expression counts for lncRNAs detected in only polyA[+] RNA libraries (red), only total RNA libraries (green) or both (gray).

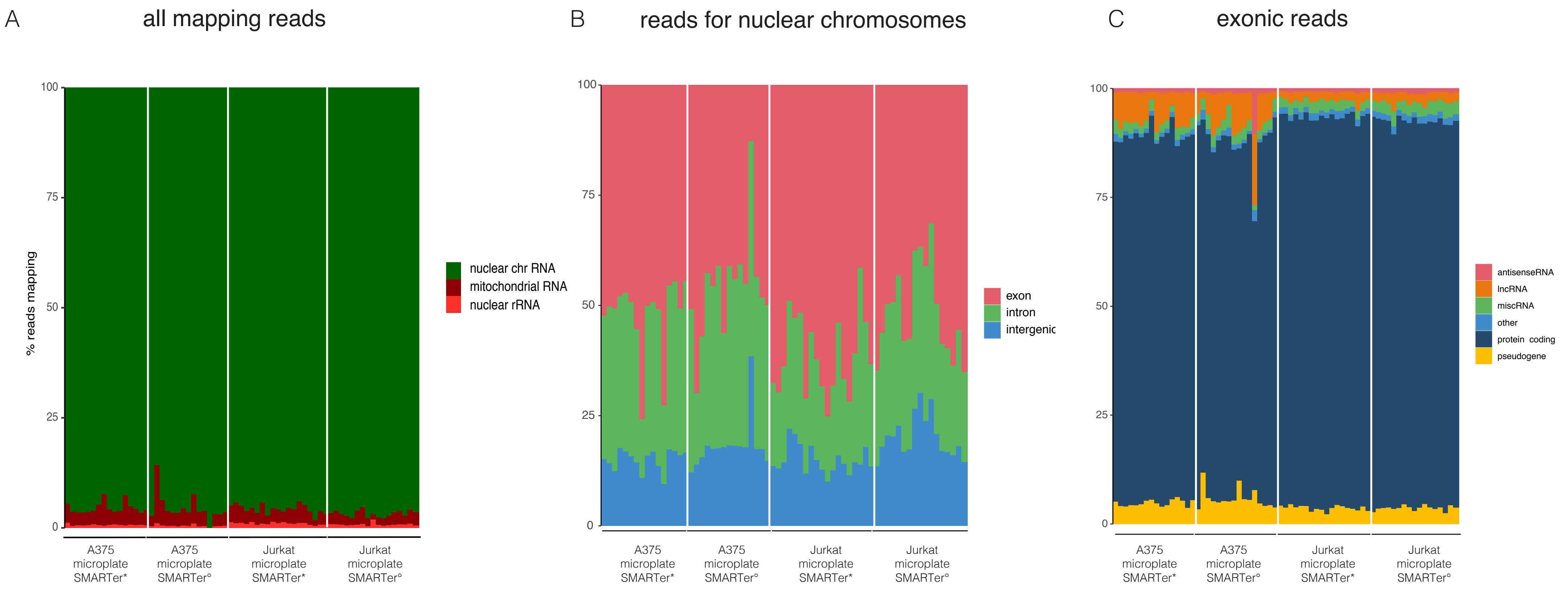

Supplementary Figure 13. SMARTer single cell total RNA sequencing works well on FACS sorted cells in microplates. Both reagents kits have similar performance (#634413, denoted with * and #634444, denoted with °; see Materials and Methods). A) Percentage of reads derived from nuclear chromosomal RNA, mitochondrial RNA and ribosomal RNA per cell quantified with STAR. B) Percentage of reads originating from nuclear chromosomes derived from exonic, intronic and intergenic regions per cell quantified with STAR. C) Percentage of exonic reads attributed to the different biotypes per cell quantified with Kallisto.
