# Supplementary material for: SMARTer single cell total RNA sequencing
Source: Nucleic Acids Res. 2019 Jun 19;47(16):e93. doi: 10.1093/nar/gkz535 (PMC6895261; doi:10.1093/nar/gkz535)
Supplement: gkz535_Supplemental_Files [file gkz535_supplemental_files.zip › Supplementary_methods.docx]

**Cell lines**

The MYCN shRNA doxycycline inducible cell line SHSY5Y-MYCN-TR is a kind gift of prof. R. Versteeg (Amsterdam, the Netherlands). The neuroblastoma cell line SK-N-BE-2C is a kind gift of prof. John Lunec (Newcastle, United Kingdom). Cells were maintained in RPMI-1640 medium (Life Technologies, 52400-025) supplemented with 10 % fetal bovine serum, 1 % of L-glutamine (Life Technologies, 15140-148) and 1 % penicillin/streptomycin (Life Technologies, 15160-047) (referred to as complete medium) at 37 °C in a 5 % CO_2_ atmosphere. Short tandem repeat (STR) genotyping was used to validate cell line authenticity prior to performing the described experiments and mycoplasma testing was done on a monthly basis.

**Cell cycle synchronization and chemical or genetic perturbation of SK-N-BE-2C and SHSY5Y-MYCN-TR cells**

SHSY5Y-MYCN-TR cells were seeded in a T75 culture flask in complete medium. After 24 hour, cells were refreshed with complete medium with either 1 µg/ml doxycycline (sigma Aldrich, D9891-1G, dissolved in ethanol) or vehicle. SK-N-BE-2C cells were synchronized as described for NGP cells. 24 hour after serum starvation, SK-N-BE-2C cells were treated with either 1 μM of JQ1 (PBS Bioscience, 27402, dissolved in DMSO) or vehicle. Cells were trypsinized 24 hour post treatment and harvested for single cell analysis and bulk RNA isolation.

**Single cell total RNA sequencing of SHSY5Y-MYCN-TR and SK-N-BE-2C cells**

Doxycycline treated SHSY5Y-MYCN-TR cells were stained with 4 µM cell tracker as described for NGP cells. An equal number of stained (doxycycline treated) and non-stained (vehicle treated) cells were mixed and diluted to 300,000 cells per ml. Suspension buffer (Fluidigm, 100-6201) was added to the cells in a 7:3 ratio and 6 µl was loaded on a primed C1 Single-Cell Open App IFC (Fluidigm, 1**00-8134**) designed for medium-sized cells (10-17 µm). Cells were captured and cDNA synthesized as described for the NGP cells with minor modifications. The reagents of the SMARTer Stranded Total RNA-Seq Kit v1 - Pico Input Mammalian (Pico v1, Takara, 635007) were used. The lysis and fragmentation were performed by incubating the cells for 3 minutes at 94 °C and 2 minutes at 10 °C and by using 9 instead of 11 PCR cycles in PCR1. Following the initial cDNA amplification, all cells were pooled in a tube using 4 µl of cDNA per cell. Next steps of the library prep were performed according to manufacturer’s instructions with minor modifications. 500 µl of 80 % ethanol was used to wash the beads. 15 PCR cycles were used for PCR2. Library quality was determined on the Bioanalyzer (Agilent).

JQ1 treated cells were stained with cell tracker as described for NGP cells. An equal number of stained (JQ1 treated) and non-stained (vehicle treated) cells were mixed and diluted to 300,000 cells per ml. Suspension buffer was added to the cells in a 8:2 ratio and 6 µl of this mix was loaded on a primed C1 Single-Cell Open App IFC designed for medium-sized cells (10-17 µm). Cells were captured and cDNA synthesized as described for the SHSY5Y-MYCN-TR cells by using the reagents of the SMARTer Stranded Total RNA-Seq Kit v2 - Pico Input Mammalian (Takara, 634413). One microliter of the ERCC spike-in mix (Ambion, 4456740) was diluted in 999 µl loading buffer to get a 1/1000 dilution of the ERCC spikes. One microliter of this dilution was added to the 20 µl lysis mix. Following the initial cDNA amplification, all cells were pooled in a tube using 5 µl of cDNA per cell. Library prep was performed as described for SHSY5Y-MYCN-TR cells.

**Single cell polyA[+] RNA sequencing of treated SHSY5Y-MYCN-TR and SK-N-BE-2C cells**

Doxycycline treated SHSY5Y-MYCN-TR cells were diluted to 300,000 cells per ml. Suspension buffer was added to the cells in a 7:3 ratio and 6 µl of this mix of was loaded on a primed C1 Single-Cell Auto Prep Array for mRNA Seq (Fluidigm, 100-6041) designed for medium-sized cells (10-17 µm) (separate IFC for treated and untreated cells). Single cell polyA[+] RNA-sequencing was performed on the C1 using the SMART-Seq v1 Ultra Low Input RNA Kit for the Fluidigm C1 System (Takara, 634833) according to manufacturer’s instructions. ArrayControl RNA spikes (Ambion, AM1780) were added as described in the manual. The concentration was measured using the quantifluor dsDNA kit (Promega, E2670) and glomax (Promega) according to manufacturer’s instructions. The samples were 1/3 diluted in C1 harvest reagent (Fluidigm). Next, library preparation was performed using the Nextera XT library prep kit (Illumina, FC-131-1096) according to manufacturer’s instructions, followed by quality control on the Bioanalyzer.

JQ1 treated cells were stained with cell tracker as described for NGP cells. An equal number of stained (JQ1 treated) and non-stained (DMSO treated) cells were mixed and diluted to 300,000 cells per ml. Suspension buffer was added to the cells in a 7:2 ratio and 6 µl of this mix of was loaded on a primed C1 Single-Cell Auto Prep Array for mRNA Seq designed for medium-sized cells (10-17 µm). Single cell polyA[+] RNA-sequencing on the C1 was performed using the SMART-Seq v1 Ultra Low Input RNA Kit for the Fluidigm C1 System (Takara) according to manufacturer’s instructions. One microliter of the ERCC spike-in mix was diluted in 999 µl loading buffer to get a 1/1000 dilution of the ERCC spikes. One microliter of this dilution was added to the 20 µl lysis mix. The quality of the cDNA was checked for 11 random single cells on the Bioanalyzer. The concentration was measured using the qubit dsDNA HS kit (Invitrogen) according to manufacturer’s instructions. The samples were 1/4 diluted in C1 harvest reagent. Next, library preparation was performed using the Nextera XT library prep kit according to manufacturer’s instructions, followed by quality control on the Bioanalyzer.

**Library sequencing**

For SHSY5Y-MYCN-TR, the polyA[+] and total RNA libraries were quantified using the KAPA library quantification kit (Roche). 1.5 pM of the total RNA library was paired-end sequenced on a NextSeq 500 (Illumina) with a read length of 36 bp and a total sequencing read depth of 347 million reads.

For the polyA[+] library, 1.2 pM of the library was paired-end sequenced on a NextSeq 500 with a read length of 75 bp and a total sequencing read depth of 250 million reads.

For SK-N-BE-2C, the polyA[+] and total RNA libraries were quantified using the KAPA library quantification kit (Roche) and libraries were diluted to 4 nM. The polyA[+] RNA library and total RNA library were pooled in a 1/2 ratio. 1.3 pM of the pooled library was single-end sequenced on a NextSeq 500 (Illumina) with a read length of 75 bp and a total sequencing read depth of 289 million reads, combining single cell polyA[+] and total RNA libraries to prevent inter-run bias.

###### Sequencing data quality control

The paired-end sequencing output of the SHSY5Y-MYCN-TR cells were trimmed using cutadapt (v.1.16) (1) to remove 3 nucleotides of the 5’ end of read 1. Remark that this changed to read 2 for the NGP version of the protocol. The SK-N-BE-2C libraries were single-end sequenced, so no trimming was needed. For both experiments, the quality of the data was assessed by mapping the reads using STAR (v.2.5.3) (2) on the hg38 genome including the full ribosomal DNA (45S, 5.8S and 5S) and mitochondrial DNA sequences. The parameters of STAR were set to retain only primary mapping reads. Using SAMtools (v.1.6) (3), reads mapping to the different nuclear chromosomes, mitochondrial DNA and rRNA were extracted and annotated as exonic, intronic or intergenic. Genes were quantified by Kallisto (v.0.43.1) (4) using both Ensembl (v.91) (5) extended with the ERCC spike sequences and LNCipedia (v.5.0) (6). The strandedness of the total RNA-seq reads was taken into account by running the –rf-stranded mode.

**Supplemental references**

1. Martin,M. (2011) Cutadapt removes adapter sequences from high-throughput sequencing reads. *EMBnet.journal*, **17**, 10.

2. Dobin,A., Davis,C.A., Schlesinger,F., Drenkow,J., Zaleski,C., Jha,S., Batut,P., Chaisson,M. and Gingeras,T.R. (2013) STAR: ultrafast universal RNA-seq aligner. *Bioinformatics*, **29**, 15–21.

3. Li,H., Handsaker,B., Wysoker,A., Fennell,T., Ruan,J., Homer,N., Marth,G., Abecasis,G. and Durbin,R. (2009) The Sequence Alignment/Map format and SAMtools. *Bioinformatics*, **25**, 2078–2079.

4. Bray,N.L., Pimentel,H., Melsted,P. and Pachter,L. (2016) Near-optimal probabilistic RNA-seq quantification. *Nat. Biotechnol.*, **34**, 525–527.

5. Zerbino,D.R., Achuthan,P., Akanni,W., Amode,M.R., Barrell,D., Bhai,J., Billis,K., Cummins,C., Gall,A., Girón,C.G., *et al.* (2018) Ensembl 2018. *Nucleic Acids Res.*, **46**, D754–D761.

6. Volders,P.J., Verheggen,K., Menschaert,G., Vandepoele,K., Martens,L., Vandesompele,J. and Mestdagh,P. (2015) An update on LNCipedia: a database for annotated human lncRNA sequences. *Nucleic Acids Res.*, **43**, 4363–4364.
